# The genome sequence of the pale mottled willow, *Caradrina clavipalpis *(Scopoli, 1763)

**DOI:** 10.12688/wellcomeopenres.18103.1

**Published:** 2022-09-12

**Authors:** Douglas Boyes, University of Oxford and Wytham Woods Genome Acquisition Lab, Darwin Tree of Life Barcoding collective, Wellcome Sanger Institute Tree of Life programme, Wellcome Sanger Institute Scientific Operations: DNA Pipelines collective, Tree of Life Core Informatics collective, Clare Boyes, Darwin Tree of Life Consortium

**Affiliations:** 1 UK Centre for Ecology and Hydrology, Wallingford, Oxfordshire, UK; 2 Independent researcher, Wytham, Oxfordshire, UK

**Keywords:** Caradrina clavipalpis, pale mottled willow, genome sequence, chromosomal, Lepidoptera

## Abstract

We present a genome assembly from an individual male *Caradrina clavipalpis *(pale mottled willow; Arthropoda; Insecta; Lepidoptera; Noctuidae). The genome sequence is 474 megabases in span. The entire assembly (100%) is scaffolded into 31 chromosomal pseudomolecules with the Z sex chromosome assembled. The complete mitochondrial genome was also assembled and is 15.6 kilobases in length.

## Species taxonomy

Eukaryota; Metazoa; Ecdysozoa; Arthropoda; Hexapoda; Insecta; Pterygota; Neoptera; Endopterygota; Lepidoptera; Glossata; Ditrysia; Noctuoidea; Noctuidae; Noctuinae; *Caradrina*; *Caradrina clavipalpis* (Scopoli, 1763) (NCBI:txid987895).

## Background

The pale mottled willow, *Caradrina clavipalpis* (Scopoli, 1763) is a widespread noctuid moth of grassland and gardens found across the western Palaearctic from Europe to Sri Lanka. It is resident in the British Isles, but it is believed that its population is boosted by immigration, as large numbers of individuals have been recorded on nights with known influxes of migrants. In Scotland and northern England, this species has declined, although its British population overall seems to be stable ( Randle *et al.,* 2019).

The adult moth is attracted to light and sugar, and also feeds at flowers. It is thought to have two generations each year in the UK, with adults on the wing in May–July and again in August–October. The adult moth is quite small with a forewing length of 12–15mm. It has mottled forewings, with a series of dashes on the leading edge. The hindwing is pearly white. 

The larvae feed on the grain of cereal crops (Graminaea) both in the field and in storage, and also plantains ( *Plantago* spp.). Historic records from coal mines described the larvae as living on the fodder of the pit-ponies ( Heath & Emmett, 1983). There are also records of the adult being infested with the mite *Cheletomorpha lepidopterum* which is found in hay bales; a previous name for this moth was the hay moth ( Forgham, 2015). Larvae pupate in autumn in a robust cocoon underground from which they emerge in spring. This early generation gives rise to the second generation later in the year ( Heath & Emmett, 1983).

The genome of *C. clavipalpis* was sequenced as part of the Darwin Tree of Life Project, a collaborative effort to sequence all of the named eukaryotic species in the Atlantic Archipelago of Britain and Ireland. Here we present a chromosomally complete genome sequence for *C. clavipalpis*, based on one ilCarClav1 specimen from Wytham Woods, Berkshire, UK.

## Genome sequence report

**Figure 1.**
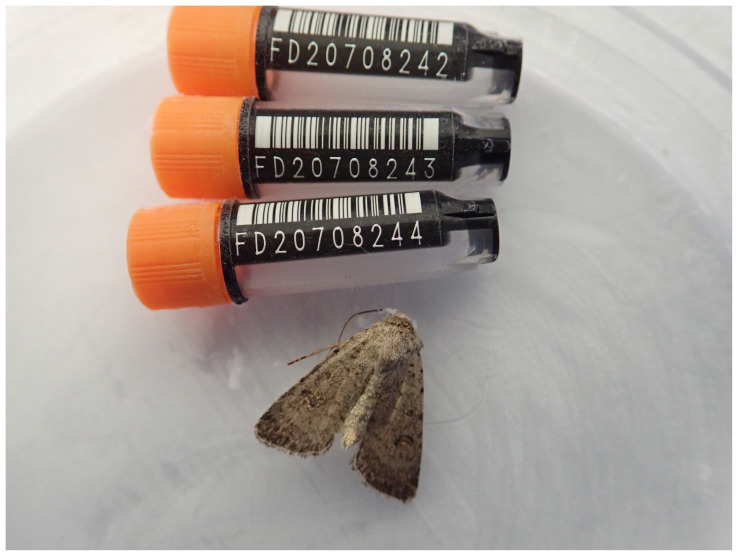
Image of the *Caradrina clavipalpis* specimen taken prior to preservation and processing.

**Figure 2.**
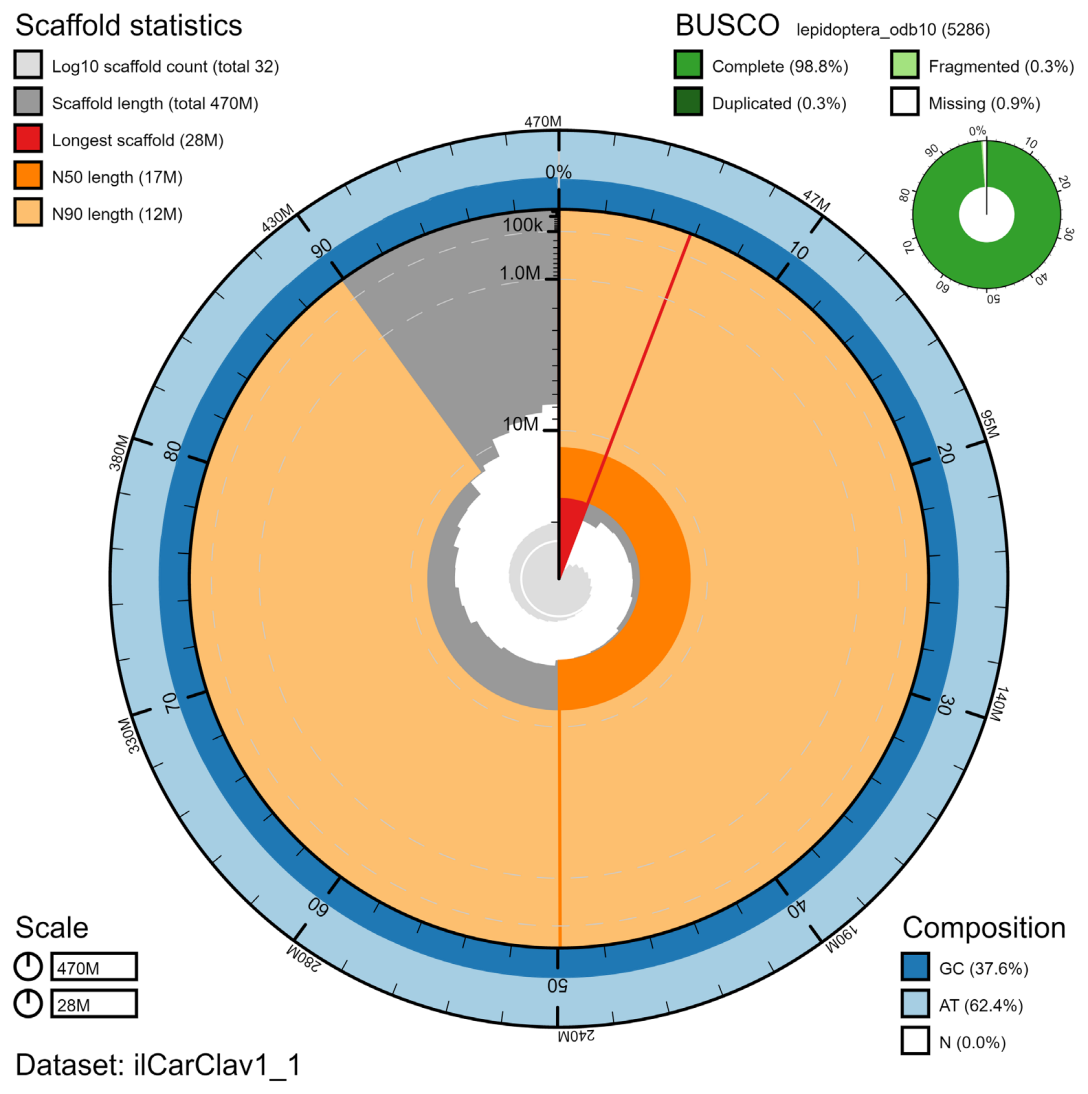
Genome assembly of *Caradrina clavipalpis*, ilCarClav1.1: metrics. The BlobToolKit Snailplot shows N50 metrics and BUSCO gene completeness. The main plot is divided into 1,000 size-ordered bins around the circumference with each bin representing 0.1% of the 474,197,171 bp assembly. The distribution of chromosome lengths is shown in dark grey with the plot radius scaled to the longest chromosome present in the assembly (27,892,075 bp, shown in red). Orange and pale-orange arcs show the N50 and N90 chromosome lengths (16,821,581 and 11,555,224 bp), respectively. The pale grey spiral shows the cumulative chromosome count on a log scale with white scale lines showing successive orders of magnitude. The blue and pale-blue area around the outside of the plot shows the distribution of GC, AT and N percentages in the same bins as the inner plot. A summary of complete, fragmented, duplicated and missing BUSCO genes in the lepidoptera_odb10 set is shown in the top right. An interactive version of this figure is available at https://blobtoolkit.genomehubs.org/view/ilCarClav1.1/dataset/ilCarClav1_1/snail.

The genome was sequenced from a single male *C. clavipalpis* collected from Wytham Woods, Berkshire, UK ( Figure 1). A total of 43-fold coverage in Pacific Biosciences single-molecule HiFi long reads and 35-fold coverage in 10X Genomics read clouds were generated. Primary assembly contigs were scaffolded with chromosome conformation Hi-C data. Manual assembly curation corrected 1 misjoin, reducing the assembly size by 0.35% and the scaffold number by 6.06%,

The final assembly has a total length of 474 Mb in 31 sequence scaffolds with a scaffold N50 of 16.8 Mb ( Table 1). The entire assembly sequence (100%) was assigned to 31 chromosomal-level scaffolds, representing 30 autosomes (numbered by sequence length) and the Z sex chromosome ( Figure 2– Figure 5; Table 2).

**Table 1.** Genome data for *Caradrina clavipalpis*, ilCarClav1.1.

*Project accession data*	
Assembly identifier	ilCarClav1.1
Species	*Caradrina clavipalpis*
Specimen	ilCarClav1 (genome assembly, Hi-C, RNA-Seq)
NCBI taxonomy ID	987895
BioProject	PRJEB50788
BioSample ID	SAMEA8603187
Isolate information	Male; thorax (genome assembly), head (Hi-C), abdomen (RNA-Seq)
*Raw data accessions*	
PacificBiosciences SEQUEL II	ERR8575392
10X Genomics Illumina	ERR8571682-ERR8571685
Hi-C Illumina	ERR8571681
PolyA RNA-Seq Illumina	ERR8571686
*Genome assembly*	
Assembly accession	GCA_932526535.1
*Accession of alternate haplotype*	GCA_932526345.1
Span (Mb)	474
Number of contigs	34
Contig N50 length (Mb)	16.8
Number of scaffolds	31
Scaffold N50 length (Mb)	16.8
Longest scaffold (Mb)	19.1
BUSCO * genome score	C:98.8%[S:98.5%,D:0.3%],F:0.3%, M:0.9%,n:5,286

*BUSCO scores based on the lepidoptera_odb10 BUSCO set using v5.3.2. C= complete [S= single copy, D=duplicated], F=fragmented, M=missing, n=number of orthologues in comparison. A full set of BUSCO scores is available at https://blobtoolkit.genomehubs.org/view/ilCarClav1.1/dataset/ilCarClav1_1/busco.

**Figure 3.**
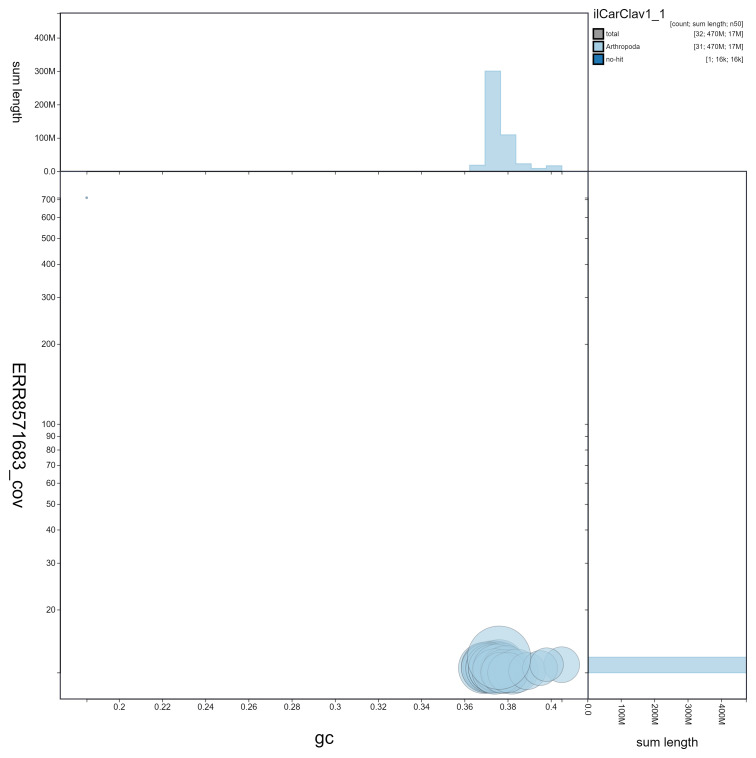
Genome assembly of *Caradrina clavipalpis*, ilCarClav1.1: GC coverage. BlobToolKit GC-coverage plot. Scaffolds are coloured by phylum. Circles are sized in proportion to scaffold length. Histograms show the distribution of scaffold length sum along each axis. An interactive version of this figure is available at https://blobtoolkit.genomehubs.org/view/ilCarClav1.1/dataset/ilCarClav1_1/blob.

**Figure 4.**
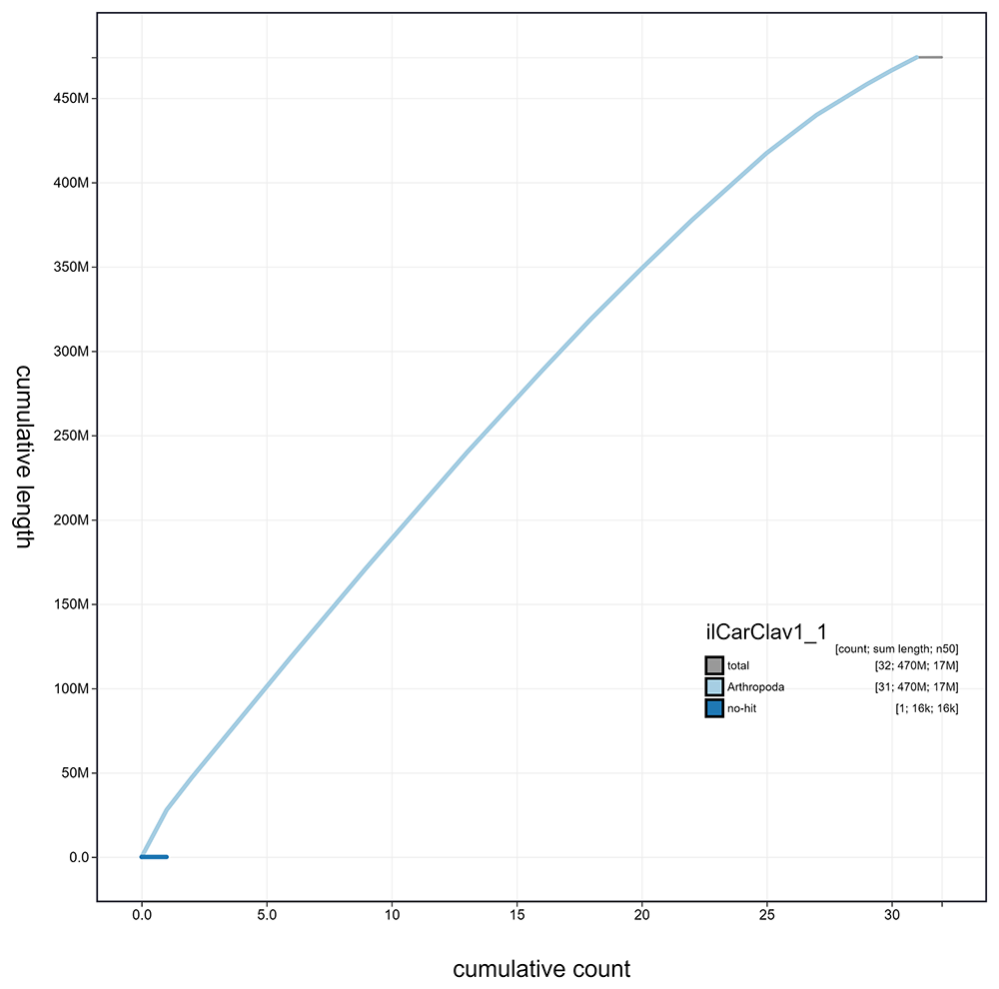
Genome assembly of *Caradrina clavipalpis*, ilCarClav1.1: cumulative sequence. BlobToolKit cumulative sequence plot. The grey line shows cumulative length for all scaffolds. Coloured lines show cumulative lengths of scaffolds assigned to each phylum using the buscogenes taxrule. An interactive version of this figure is available at https://blobtoolkit.genomehubs.org/view/ilCarClav1.1/dataset/ilCarClav1_1/cumulative.

**Figure 5.**
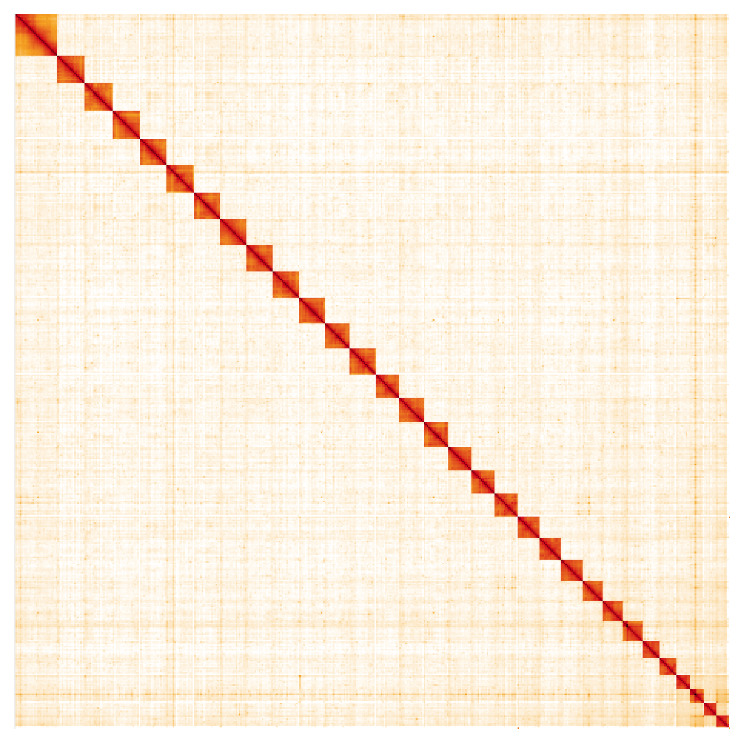
Genome assembly of *Caradrina clavipalpis*, ilCarClav1.1: Hi-C contact map. Hi-C contact map of the ilCarClav1.1 assembly, visualised in HiGlass. Chromosomes are arranged in size order from left to right and top to bottom. The interactive Hi-C map can be viewed at https://genome-note-higlass.tol.sanger.ac.uk/l/?d=SLhcGGQnQYuPR-bMu03f2Q.

**Table 2.** Chromosomal pseudomolecules in the genome assembly of *Caradrina clavipalpis*, ilCarClav1.1.

INSDC accession	Chromosome	Size (Mb)	GC%
OW052088.1	1	19.12	37.3
OW052089.1	2	18.16	37.6
OW052090.1	3	18.02	36.9
OW052091.1	4	17.99	37.6
OW052092.1	5	17.79	37.1
OW052093.1	6	17.74	37.4
OW052094.1	7	17.5	37.4
OW052095.1	8	17.48	37.1
OW052096.1	9	17.13	37
OW052097.1	10	17.07	37.2
OW052098.1	11	16.97	37
OW052099.1	12	16.82	37.2
OW052100.1	13	16.19	37.3
OW052101.1	14	16.14	37.3
OW052102.1	15	16.1	37.5
OW052103.1	16	15.74	37.7
OW052104.1	17	15.59	37.5
OW052105.1	18	14.88	37.8
OW052106.1	19	14.66	37.9
OW052107.1	20	14.29	37.9
OW052108.1	21	14.24	37.4
OW052109.1	22	13.63	38.2
OW052110.1	23	13.2	37.8
OW052111.1	24	13.1	38.4
OW052112.1	25	11.56	37.7
OW052113.1	26	11.05	38
OW052114.1	27	9.38	38.9
OW052115.1	28	8.77	40.5
OW052116.1	29	8.31	39.5
OW052117.1	30	7.69	39.8
OW052087.1	Z	27.89	37.6
OW052118.1	MT	0.02	18.6

The assembly has a BUSCO v5.3.2 ( Manni *et al.,* 2021) completeness of 98.8% (single 98.5%, duplicated 0.3%) using the lepidoptera_odb10 reference set (n=5,286). While not fully phased, the assembly deposited is of one haplotype. Contigs corresponding to the second haplotype have also been deposited.

## Methods

### Sample acquisition and nucleic acid extraction

A single male *C. clavipalpis* specimen (ilCarClav1) was collected using a light trap from Wytham Woods, Berkshire, UK (latitude 51.772, longitude -1.338) by Douglas Boyes (University of Oxford). The specimen was identified by Douglas Boyes and snap-frozen on dry ice. 

DNA was extracted at the Tree of Life laboratory, Wellcome Sanger Institute. The ilCarClav1 sample was weighed and dissected on dry ice with head tissue set aside for Hi-C sequencing. Thorax tissue was disrupted using a Nippi Powermasher fitted with a BioMasher pestle. Fragment size analysis of 0.01–0.5 ng of DNA was then performed using an Agilent FemtoPulse. High molecular weight (HMW) DNA was extracted using the Qiagen MagAttract HMW DNA extraction kit. Low molecular weight DNA was removed from a 200-ng aliquot of extracted DNA using 0.8X AMpure XP purification kit prior to 10X Chromium sequencing; a minimum of 50 ng DNA was submitted for 10X sequencing. HMW DNA was sheared into an average fragment size between 12–20 kb in a Megaruptor 3 system with speed setting 30. Sheared DNA was purified by solid-phase reversible immobilisation using AMPure PB beads with a 1.8X ratio of beads to sample to remove the shorter fragments and concentrate the DNA sample. The concentration of the sheared and purified DNA was assessed using a Nanodrop spectrophotometer and Qubit Fluorometer and Qubit dsDNA High Sensitivity Assay kit. Fragment size distribution was evaluated by running the sample on the FemtoPulse system.

RNA was extracted from abdomen tissue of ilCarClav1 in the Tree of Life Laboratory at the WSI using TRIzol, according to the manufacturer’s instructions. RNA was then eluted in 50 μl RNAse-free water and its concentration RNA assessed using a Nanodrop spectrophotometer and Qubit Fluorometer using the Qubit RNA Broad-Range (BR) Assay kit. Analysis of the integrity of the RNA was done using Agilent RNA 6000 Pico Kit and Eukaryotic Total RNA assay.

### Sequencing

Pacific Biosciences HiFi circular consensus and 10X Genomics Chromium read cloud sequencing libraries were constructed according to the manufacturers’ instructions. Sequencing was performed by the Scientific Operations core at the Wellcome Sanger Institute on Pacific Biosciences SEQUEL II (HiFi), Illumina NovaSeq 6000 (10X) and Illumina HiSeq 4000 (RNA-Seq) instruments. Hi-C data were generated in the Tree of Life laboratory from head tissue of ilCarClav1 using the Arima v2 kit and sequenced on a NovaSeq 6000 instrument.

### Genome assembly

Assembly was carried out with Hifiasm ( Cheng *et al.,* 2021); haplotypic duplication was identified and removed with purge_dups ( Guan *et al.,* 2020). One round of polishing was performed by aligning 10X Genomics read data to the assembly with longranger align, calling variants with freebayes ( Garrison & Marth, 2012). The assembly was then scaffolded with Hi-C data ( Rao *et al.,* 2014) using YaHS ( Zhou *et al.,* 2022). The assembly was checked for contamination as described previously ( Howe *et al.,* 2021). Manual curation was performed using HiGlass ( Kerpedjiev *et al.,* 2018) and Pretext. The mitochondrial genome was assembled using MitoHiFi ( Uliano-Silva *et al.,* 2021), which performs annotation using MitoFinder ( Allio *et al.,* 2020). The genome was analysed and BUSCO scores generated within the BlobToolKit environment ( Challis *et al.,* 2020). Table 3 contains a list of all software tool versions used, where appropriate.

**Table 3.** Software tools used.

Software tool	Version	Source
Hifiasm	0.15.3	Cheng *et al.,* 2021
purge_dups	1.2.3	Guan *et al.,* 2020
YaHS	1.0	Zhou *et al.,* 2022
longranger align	2.2.2	https://support.10xgenomics.com/ genome-exome/software/pipelines/ latest/advanced/other-pipelines
freebayes	1.3.1-17- gaa2ace8	Garrison & Marth, 2012
MitoHiFi	2.0	Uliano-Silva *et al.,* 2021
HiGlass	1.11.6	Kerpedjiev *et al.,* 2018
PretextView	0.2.x	https://github.com/wtsi-hpag/ PretextView
BlobToolKit	3.2.6	Challis *et al.,* 2020

### Ethics/compliance issues

The materials that have contributed to this genome note have been supplied by a Darwin Tree of Life Partner. The submission of materials by a Darwin Tree of Life Partner is subject to the Darwin Tree of Life Project Sampling Code of Practice. By agreeing with and signing up to the Sampling Code of Practice, the Darwin Tree of Life Partner agrees they will meet the legal and ethical requirements and standards set out within this document in respect of all samples acquired for, and supplied to, the Darwin Tree of Life Project. Each transfer of samples is further undertaken according to a Research Collaboration Agreement or Material Transfer Agreement entered into by the Darwin Tree of Life Partner, Genome Research Limited (operating as the Wellcome Sanger Institute), and in some circumstances other Darwin Tree of Life collaborators.

## Data availability

European Nucleotide Archive: Caradrina clavipalpis (pale mottled willow). Accession number PRJEB50788; https://identifiers.org/ena.embl/PRJEB50788.

The genome sequence is released openly for reuse. The *C. clavipalpis* genome sequencing initiative is part of the Darwin Tree of Life (DToL) project. All raw sequence data and the assembly have been deposited in INSDC databases. The genome will be annotated using the RNA-Seq data and presented through the Ensembl pipeline at the European Bioinformatics Institute. Raw data and assembly accession identifiers are reported in Table 1.

## Author information

Members of the University of Oxford and Wytham Woods Genome Acquisition Lab are listed here: https://doi.org/10.5281/zenodo.6418202.

Members of the Darwin Tree of Life Barcoding collective are listed here: https://doi.org/10.5281/zenodo.6418156.

Members of the Wellcome Sanger Institute Tree of Life programme are listed here: https://doi.org/10.5281/zenodo.6866293.

Members of Wellcome Sanger Institute Scientific Operations: DNA Pipelines collective are listed here: https://doi.org/10.5281/zenodo.5746904.

Members of the Tree of Life Core Informatics collective are listed here: https://doi.org/10.5281/zenodo.6125046.

Members of the Darwin Tree of Life Consortium are listed here: https://doi.org/10.5281/zenodo.6418363.
