# Supplementary material for: The genome sequence of the pale mottled willow, Caradrina clavipalpis (Scopoli, 1763)
Source: Wellcome Open Res. 2022 Sep 12;7:225. [Version 1] doi: 10.12688/wellcomeopenres.18103.1 (PMC10043633; doi:10.12688/wellcomeopenres.18103.1)
Supplement: Peer review history

# Peer review: Reviewer response for version 1

**DOI:** 10.21956/wellcomeopenres.20074.r55376

**Published:** 2023-04-11

**Authors:** Yu-Shin Nai

**Affiliations:** 1 National Chung Hsing University,, Taichung City, Taiwan

This genomic sequencing report is well-organized. I have only two suggestions:

1. How to validate the real chromosome number? The karyotype of the *Caradrina clavipalpis *might be conducted to confirm this point.
2. In the "Methods" section, the author mentioned RNA extraction, but the purpose is not clearly described. Besides, I didn't see the summary of any RNA-seq result or annotation table. Therefore, the authors should carefully check this part.

Are sufficient details of methods and materials provided to allow replication by others?

Partly

Is the rationale for creating the dataset(s) clearly described?

Partly

Are the datasets clearly presented in a useable and accessible format?

Yes

Are the protocols appropriate and is the work technically sound?

Yes

Reviewer Expertise:

Insect Pathology, Microbiology, Genomics

I confirm that I have read this submission and believe that I have an appropriate level of expertise to confirm that it is of an acceptable scientific standard.

# Peer review: Reviewer response for version 1

**DOI:** 10.21956/wellcomeopenres.20074.r55371

**Published:** 2023-03-27

**Authors:** Xueyan Li

**Affiliations:** 1 Chinese Academy of Sciences,, Kunming, China

This data presents a high-quality and integrity genome assembly of a pale mottled willow, *Caradrina Clavipalpis*, which updates the genome repository for Lepidoptera. The data provide a genetic foundation for further studies on the systematics and complex phenotypic evolution of moths even Lepidoptera. Some problems are worthy of further clarification:

1. Table 1 provides PolyA RNA-Seq Illumina data and the article also mentions, providing annotation results for this genome will be more conducive to the extensive use of the data.
2. The abstract mentions the mitochondrial genome, but there are no corresponding results in the main text.
3. The methods section needs to be more detailed. For example, please provide the reagent kit or method used for DNA extraction, and important parameters for genome assembly software.

Are sufficient details of methods and materials provided to allow replication by others?

Yes

Is the rationale for creating the dataset(s) clearly described?

Yes

Are the datasets clearly presented in a useable and accessible format?

Yes

Are the protocols appropriate and is the work technically sound?

Yes

Reviewer Expertise:

Insect genome

I confirm that I have read this submission and believe that I have an appropriate level of expertise to confirm that it is of an acceptable scientific standard.

# Peer review: Reviewer response for version 1

**DOI:** 10.21956/wellcomeopenres.20074.r52736

**Published:** 2022-10-25

**Authors:** Martin Pippel

**Affiliations:** 1 Max Planck Institute of Molecular Cell Biology and Genetics, Dresden, Germany

“The genome sequence of the pale mottled willow, *Caradrina clavipalpis*” from Boyes *et al. *describes a high-quality genome assembly based on PacBio HiFi reads, 10X Genomics read clouds and HiC data. 

 The data note is well structured and based on the described methods and the publicly available sequencing data also reproducible by the scientific community. The primary assembly has a very high contiguity already at the contig level. Only two scaffolds consist of more than one contig and most of them do have the telomer sequence motif at both ends.

 Methods:

 Genome assembly methods are a bit short and it could be hard to reproduce the assembly without further documentation of the used program arguments. Even if all tools were run in default mode, it would be worth mentioning. Some of the used tools, such as purge_dups depend itself on other programs like minimap2. But those could not be found in Table 3. I could not find any information if all variants from Freebayes were used for the error polishing or if a variant filtering step was applied beforehand.

 I do only have some minor comments and suggestions:

- I could not find the HiC read coverage in the Data Note
- the plots from figure 3 and 4 are for such a high-contiguous assembly not very informative
- additional statistics about the sequencing data (e.g. read length N50 of HiFi reads and 10X read clouds, kmer based genome size and heterozygosity estimates) and the final assembly (e.g. merqury QV scores, repeat content) would be nice to see as well
- error-polishing strategies of HiFi-based assemblies are currently under debate. I assume you applied bcftools consensus on the filtered Freebayes VCF files similar to the VGP assembly pipeline ( https://github.com/VGP/vgp-assembly/tree/master/pipeline). How big was the improvement based in the QV-score? Were the alternate contigs included in the longranger alignment step?
- RNAseq was produced but it was not used for an annotation, why?
- It is great that you also included the ccs bam file, including the kinetics information. A further great feature would be to make the potential methylation sites (5mc in CpG islands) available for both assemblies

Are sufficient details of methods and materials provided to allow replication by others?

Yes

Is the rationale for creating the dataset(s) clearly described?

Yes

Are the datasets clearly presented in a useable and accessible format?

Yes

Are the protocols appropriate and is the work technically sound?

Yes

Reviewer Expertise:

Genome assembly and annotation, population genomics

I confirm that I have read this submission and believe that I have an appropriate level of expertise to confirm that it is of an acceptable scientific standard.
